# Etiology of Pericardial Effusion and Outcomes Post Pericardiocentesis in the Western Region of Saudi Arabia: A Single-center Experience

**DOI:** 10.7759/cureus.6627

**Published:** 2020-01-11

**Authors:** Saad Albugami, Faisal Al-Husayni, Abdullah AlMalki, Mohammed Dumyati, Ysear Zakri, Jamilah AlRahimi

**Affiliations:** 1 Cardiology, King Faisal Cardiac Center, King Saud Bin Abdulaziz University for Health Sciences, King Abdullah International Research Center, Jeddah, SAU; 2 Internal Medicine, National Guard Hospital, Jeddah, SAU; 3 Internal Medicine, National Guard Health Affairs, King Abdulaziz Medical City, Jeddah, SAU

**Keywords:** pericardiocentesis, pericardial diseases, pericardial effusion, cardiovascular diseases

## Abstract

Background

Pericardial effusion is the accumulation of blood or excess fluid in the cavity between the heart and the pericardium sac. Pericardial effusion can be caused by several etiologies, including malignant and non-malignant causes. Pericardiocentesis is the gold standard assessment method for pericardial effusion etiology. The aim of this study was to identify the long-term outcome of patients who presented with massive pericardial effusion and underwent pericardiocentesis at King Abdulaziz Medical City, Jeddah, a large tertiary hospital in the western part of Saudi Arabia.

Methods

This is a single-center retrospective cross-sectional study conducted at King Abdulaziz Medical City Jeddah, Saudi Arabia, between January 2013 to December 2018. Data were collected from patient’s charts; the clinical and echocardiographic findings, alongside with pericardial fluid analysis, were collected. Procedure and patients outcomes were obtained and reported.

Results

Of the 107 patients with pericardial effusion, 39 patients had moderate to severe pericardial effusion requiring pericardiocentesis. The mean age was 52 years, and 56.4% were females. The most common chronic disease was hypertension and the presence of metastasis. The most common cause of pericardial effusion was a malignancy. A majority of patients had severe pericardial effusion. Many patients had tamponade (69.6%). Patients with malignant pericardial effusion had a median survival of 54 days.

Conclusion

Etiologies of pericardial effusion requiring drainage depend on the population studied. Patients with malignant effusions have worse outcomes than non-malignant effusion. Pericardiocentesis is required to ascertain the cause and risk-stratify patients.

## Introduction

The normal pericardium is a double-layered sac that encircles the heart and roots of the large vessels. It is composed of two different layers; the outer one is the fibrous parietal pericardium, whereas, the inner one is the visceral pericardium [1]. The pericardium prevents the displacement of the heart and large vessels, prevents sudden dilatation of the heart, and the spread of infection or cancer from the pleura or lung as well as minimizes friction between the heart and surrounding structures [2]. The pericardial cavity is located between the parietal and visceral pericardium, and it is filled with 10-50 cc of fluid, which is ultrafiltrate of plasma and produced by the visceral pericardium. This fluid acts as a lubricant between the pericardium and the heart; however, blood accumulation and excess fluid in this cavity is called pericardial effusion [3-4]. The symptoms of pericardial effusion include cough, chest pain, dyspnea, and orthopnea [5]. Pericardial effusion develops in patients with diseases that affect the pericardium such as systemic disorders and pericarditis [1,4]. Pericardial effusion can be attributed to several etiologies, including malignant and non-malignant causes [6]. The known causes include neoplasia, infection, congestive heart failure, Iatrogenicity, radiation, trauma, connective tissue diseases, pericardial injury, and metabolic causes such as uremia and hypothyroidism; a substantial number of effusions are idiopathic [7-9]. 

The exact cause of pericardial effusion can be identified by pericardiocentesis, which is indicated when the effusion is symptomatic or significant [2]. It is also indicated when the effusion is accompanied by tamponade, or the cause of the effusion is uncertain [2]. Pericardiocentesis is the gold-standard method to ascertain the etiology of pericardial effusion [10]. This study was conducted to identify the etiology of pericardial effusion as well as the intermediate-term outcome of patients who underwent pericardiocentesis at King Abdulaziz Medical City in Jeddah, Saudi Arabia. 

## Materials and methods

This research is an observational cross-sectional study conducted at King Abdulaziz Medical City, Jeddah. The study included all patients who underwent percutaneous pericardiocentesis between January 2013 till December 2018. Patients who had surgical drainage or were less than the age of 16 years were excluded. The data were collected from patients‘ files, including sex, age, date of procedure, medical history, laboratory values, effusion size, fluid sample characteristics, clinical diagnosis, and date of death if available. Results of serological testing, if reported, cultures in peripheral blood, and pericardial fluid results were obtained. Effusion size was determined by reviewing pre-procedural echocardiogram reports. The small size was defined as <10 mm, medium >10 and <20 mm, and large >20 mm. Analyses of fluid characteristics, including macroscopic aspects, biochemistry, cytology, and microbiology, were collected. 

Statistical analysis

Statistical analysis was conducted using STATA 12 software (StataCorp LP, TX). Continuous variables were presented as mean, standard deviation. Inter-group differences were compared using the t-test. Skewed numerical data were presented as median and average rank, and between-group differences were compared using the Mann-Whitney U test. Paired numerical data were compared using the paired t-test. Categorical variables were presented as number and percentage, and differences between groups were compared using the Pearson chi-squared test or Fisher’s exact test. Ordinal data were compared using the chi-squared test for trend. Paired binary data were compared using the McNemar test, and paired ordinal data using the Stuart-Maxwell test of marginal homogeneity; p-values <0.05 were considered statistically significant.

## Results

The hospital electronic system identified 107 patients with pericardial effusion. Among those, only 39 met the inclusion criteria. All patients had been diagnosed with pericardial effusion using echocardiography and were categorized into mild, moderate, and severe. The mean age was 52 ± 19 years old. Females represented more than half of patients 22 (56.4%). Comorbid diseases were reported as follows; 28.2% had diabetes mellitus, 35.9% had hypertension, while heart failure was seen in 18%. Twenty percent had lung cancer; breast cancer was seen in 15.4% and lymphoma in 7.7%. Metastasis was present among 35.9%. Five patients (12.8%) had autoimmune diseases, and one patient (2.6%) was not known to have any medical illness. Patients‘ demographics and chronic diseases are shown in Table 1.

**Table 1:** Demographics and chronic diseases of pericardial effusion patients

Variable	Frequency	%
Mean Age ± SD (Median)	52 ± 19 (54)	
Gender		
Male	17	43.6
Female	22	56.4
Chronic diseases		
Diabetes mellitus	11	28.2
Hypertension	14	35.9
Chronic kidney disease	8	20.5
Dyslipidemia	1	2.6
Heart failure	7	18
Lung cancer	8	20.5
Breast cancer	6	15.4
Lymphoma	3	7.7
Multiple myeloma	1	2.6
Prostate cancer	1	2.6
Leukemia	1	2.6
Metastasis	14	35.9
Autoimmune diseases	5	12.8
No medical illness	1	2.6

The most common causes of pericardial effusion were malignancy (48.7%), followed by infection, and uremia; both accounted for 15.4%. (Table 2). Iatrogenic causes of pericardial effusion requiring pericardiocentesis were as follows: three cases (7.6%) post-cardiac surgery and one case post arrhythmia ablation. Bacterial infection was the predominant cause of infected pericardial effusion (15.4%).

**Table 2:** Etiology of pericardial effusion

Cause	Frequency	%
Idiopathic	4	10.3
Malignancy	19	48.7
Iatrogenic	4	10.3
Infection	6	15.4
Heart failure	3	7.7
Uremia	6	15.4
Autoimmune	2	5.1

There were 32 cases of severe pericardial effusion (82%); 27 of them (69.2%) had echocardiographic features of cardiac tamponade (Table 3).

**Table 3:** Pericardial effusion severity for patients who underwent pericardiocentesis

Severity	Frequency	%
Mild	0	0
Moderate	7	18
Severe	32	82
Cardiac Tamponade		
Yes	27	69.2
No	12	30.8

The pericardial fluid appearance was bloody in 25 patients (64.1%), while serous and serosanguinous appearances were 25.5% and 15.4%, respectively. Eighteen percent showed malignant cytology. Pericardial fluid analysis is shown in Table 4.

**Table 4:** Pericardial fluid analysis

Appearance	Frequency	%
Bloody	25	64.1
Serous	8	25.5
Serosanguinous	6	15.4
Cytology		
Malignant	7	18
Normal	32	82

Following pericardiocentesis, medium-term outcomes are demonstrated in Table 5. Nineteen patients (50%) died within two months. Re-accumulation was seen in 20.5% of the patients that required re-drainage. Procedure-related complications like arrhythmia, hematoma, and cardiac arrest were seen in 5.1%, 5.1%, and 2.6%, respectively.

**Table 5:** Outcome after pericardiocentesis

Outcome	Frequency	%
Re-accumulation	8	20.5
Repeated pericardiocentesis	3	7.7
Arrhythmia	2	5.1
Hematoma	2	5.1
Arrest	1	2.6
Hypotension	2	5.1
Death	19	50

There was a significant association between malignant pericardial effusion and death (P-value 0.0001) (Table 6).

**Table 6:** Correlation between malignancy and outcomes after pericardiocentesis

Outcome	Chi2	P value
Death	15.2	0.0001
Re-accumulation	0.76	0.38
Repeated pericardiocentesis	0.41	0.51
Arrhythmia	0.0014	0.97
Hematoma	2	0.15
Arrest	1	0.29
Hypotension	2.2	0.136

**Figure 1:**
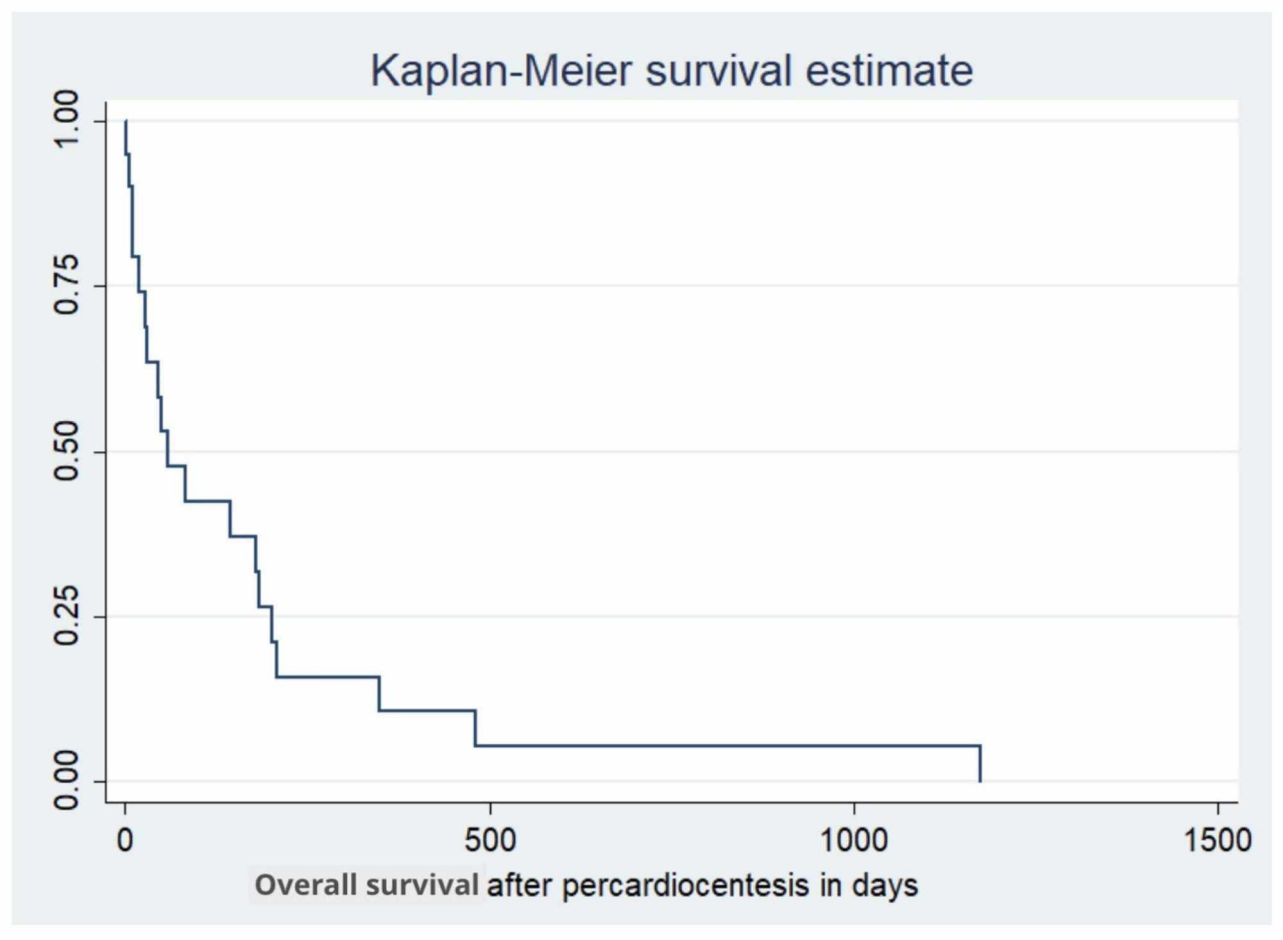
Kaplan-Meier curve showing overall survival of pateints following pericardiocentesis, with median of 54 days

The median survival of patients with pericardial effusion post drainage was reported to be 54 days, as shown in Figure 1.

## Discussion

Uremic and tuberculous pericarditis were reported previously to be the most common causes of pericardial disease in the southern part of Saudi Arabia (Asir region), which may probably reflect the spectrum of diseases prevailing in that area [11]. Specific cause dominance of pericardial effusion depends on the population characteristics under study as well as the function of the healthcare facility they present to [12]. Historically, both malignancy and uremia were considered to be the most common causes of pericardial effusions [13]. Colombo et al. described 20 patients with pericardial effusion, 44% have presented with cardiac tamponade. Neoplastic (44%), idiopathic (32%), and uremia (20%) were found to be the main reasons that cause cardiac tamponade [14]. Turak et al. described 104 patients with established moderate to severe pericardial effusion; idiopathic conditions were found to be the leading cause of pericardial effusions [15]. They also showed that malignancy, congestive heart failure, and tuberculosis were other primary etiologies that might lead to pericardial effusion. In another large study that consisted of 322 patients, 132 patients had moderate and 190 patients had severe pericardial effusion. Among them, the prevalence of cardiac tamponade was found to be 37%. In that study, idiopathic (16%), iatrogenic (16%), and neoplastic conditions (13%) were designated as common causes of pericardial effusion [16]. The current study was conducted on 39 patients from the western coast of Saudi Arabia. The most prevalent cause of pericardial effusion among these patients was malignancy (48.7%). In contrast, infection and uremia were ranked second. This is probably because the hospital is a tertiary care center for oncology patients and has a large dialysis unit. Also, all infectious effusions were secondary to bacteria. Iatrogenic and idiopathic causes had the same prevalence of 10.3%. There were 82% of patients with severe effusion; 69% of them had tamponade. The median overall survival was 54 days. It is not surprising that patients with malignant effusion had significantly worse survival. Our data is in agreement with what was reported by Strobbe et al. and El Haddad et al., both reported a worse survival among cancer patients [6,17]. Our report is the second one from Saudi Arabia, albeit from a different geographical area. It shows different results to what was reported previously, reflecting the divergent frequency of the underlying diseases from a different population.

Limitations

Our is a single-center, retrospective cross-sectional study. Certain variables, such as serological tests or clinical characteristics were not systematically recorded. Higher percentage of patients with cancer was included likely because the hospital has a large oncology tertiary care centre. Our patient population might not necessarily be representative of the region population due to selection bias. Besides, the total number was relatively small.

## Conclusions

The majority of patients with large pericardial effusions necessitating drainage, in our study, are due to cancer. Malignant pericardial effusion carries a poor long-term prognosis. Extensive multicentre studies are required to compare populations, and ascertain causes relevant to each geographical area.
